# Epidemiologic Profile of Patients With Epilepsy in a Region of Southeast Brazil: Data From a Referral Center

**DOI:** 10.3389/fneur.2022.822537

**Published:** 2022-05-10

**Authors:** Renata Parissi Buainain, Carlos Tadeu Parisi Oliveira, Fernando Augusto Lima Marson, Manoela Marques Ortega

**Affiliations:** ^1^Laboratory of Cell and Molecular Tumor Biology and Bioactive Compounds, São Francisco University, São Paulo, Brazil; ^2^Laboratory of Human and Medical Genetics, São Francisco University, São Paulo, Brazil; ^3^São Francisco University Hospital, São Paulo, Brazil

**Keywords:** epilepsy, epidemiology, prevalence, neurocysticercosis, neuroinfection, traumatic brain injury

## Abstract

**Introduction:**

Epilepsy affects about 50 million people worldwide, 80% of whom live in low- and middle-income countries. In Brazil, epidemiological studies are outdated and restricted to specific regions, mostly due to the continental size of country.

**Objective:**

We aimed to present the first evidence-based study on the epidemiological aspects of individuals with epilepsy, mapping the characteristics of this disease in a referral center in a region of Southeast Brazil.

**Methods:**

A retrospective study was carried out from January 2010 to March 2021. Patients were selected according to the International League Against Epilepsy Criteria.

**Results:**

From a total of 618 selected patients, 317 (51.3%) were men and 301 (48.7%) were women with an average age of 34.03 ± 20.66 years. The average age at the first seizure was 15.16 ± 17.61 years. The prevalence ratio was 1.30 cases/1,000 habitants. Childhood febrile seizure was present in 44 patients (7.9%) and family history of epilepsy in 231 (37.4%) patients. The predominant type of seizure was focal in 401 (64.9%) patients. The most frequent etiologies were structural in 254 (41.1%) patients and unknown in 238 (38.5%) patients. Most of the patients' treatments were based on anti-seizure drugs in monotherapy [389 (62.9%)] with 398 (64.4%) drug-responsive patients.

**Conclusions:**

Our epilepsy prevalence rate was lower than other studies in the Southeast Region of Brazil. In addition, the structural epilepsy type was predominant in our study compared with unknown causes, which is more frequent in other Brazilian regions and worldwide studies. The differences may be attributed to our region, which presents a high prevalence of neuroinfection, specially neurocysticercosis, and a referral center for traumatic brain injury. Moreover, the contrasting results reinforce the need for an adequate epidemiological assessment of epilepsy incidence in a region of Southeast Brazil.

## Key points

- In line with previous Brazilian studies, in our study we found similar race (white prevalence), the average age of 34.06 ± 20.66 years, average age at seizures' onset of 15.16 ± 17.61 years, the presence of childhood febrile seizures (7.9%), focal type seizures (64.9%), monotherapy treatment (62.9%), and drug-responsiveness (64.4%).
- Our epilepsy prevalence rate was lower than other studies in the Southeast Region of Brazil, which reinforces the need for an adequate epidemiological assessment of epilepsy incidence in Southeast Brazil, the most populated region.
- The most frequent etiology was structural (41.1%) compared with previous Brazilian and worldwide studies, where unknown (up to 50.0%) was more prevalent. The difference between etiologies can be attributed to the high prevalence of neuroinfection as neurocysticercosis and due to our hospital being a referral center for traumatic brain injury.
- Our study population presented a prevalence ratio of 1.3 cases/1,000 habitants.

## Introduction

Epilepsy is a chronic condition characterized by unprovoked recurrent epileptic seizures (1) and may affect the individuals of all ages, sexes, races, income groups, and geographical areas (2). Epilepsy affects about 50 million people worldwide, 80% of whom live in low- and middle-income countries (2). In 2017, the prevalence rate of epilepsy in developing countries was 8.75 per 1,000 individuals and in developed countries was 5.18 per 1,000 individuals (3).

The prevalence rate of epilepsy in developing countries is usually higher than in developed countries, mostly due to higher neurocysticercosis and head trauma rates and the lack of available treatments (4). In addition, despite the very-low cost of effective anti-seizure drugs, more than 75% of people with epilepsy in low-income countries do not receive any treatment (2). The Global Burden of Disease Study of Epilepsy (GBDE) estimated over 125,000 deaths associated with epilepsy in 2016, of which 81% were in low- and middle-sociodemographic index countries (5).

In fact, between 1990 and 2016, there was a non-significant change in the age-standardized prevalence of idiopathic epilepsy. In contrast, a significant decrease in age-standardized mortality rates and age-standardized disability-adjusted life-years rates were observed (5). Thus, to reduce the rate of epileptic seizures in low-income countries, it would be necessary to facilitate access to the anti-seizure treatments and effective new drugs development (5).

Garcia-Martin and Serrano-Castro (6) conducted an epilepsy epidemiological review study in Spain and Latin America due to the similarity of the population. Despite changes in the economic development and health conditions, the authors concluded that there was no evidence of any changes in the epidemiology of epilepsy in Spain and Latin America in the last 15 years. In addition, Bolivia, Colombia, Ecuador, and Peru, known as the endemic areas of cysticercosis, showed the highest prevalence and incidence rates of epilepsy (6).

To the best of our knowledge, only 11 epidemiological epilepsy population studies have been conducted in Brazil between 1986 and 2018 (7–17) (Table 1). In Brazil, epidemiological studies are rare besides being outdated and restricted to specific regions due to the continental size of country, difficulty in collaboration between the referral centers, and diagnostic errors. This study aimed to present the prevalence of epilepsy in a region of Southeast Brazil, describing the patients' characteristics of this disease in the referral center as the first evidence-based epidemiological aspect in this Brazilian region.

**Table 1:** Prevalence of epilepsy in epidemiological studies in Brazil between 1986 and 2021.

**City—state**	***N***	**Prevalence of epilepsy**	**Prevalence of active epilepsy**	**Age group**	**References**
São Paulo/São Paulo (urban area)	7,603 interviews	11.9/1,000	—	All ages	(7)
Florianópolis/Santa Catarina (epilepsy clinic)	120 medical records	294.8/1,000	—	>18 years	(8)
Paranatinga-Nobres/Mato Grosso (Indigenous Bakairi)	483 interviews	186/1,000	124/1,000	All ages	(9)
Rio de Janeiro/Rio de Janeiro (urban community)	982 interviews	16.3/1,000	5.1/1,000	All ages	(10)
São José do Rio Preto/São Paulo	17,293 interviews	18.6/1,000	8.2/1,000	All ages	(11)
São José do Norte/Rio Grande do Sul (rural and urban areas)	531 interviews	45.2/1,000	—	<5 years	(12)
Districts of Barão Geraldo-Jaguaré- Santo Antônio/São Paulo	54,102 interviews	9.2/1,000	5.4/1,000	All ages	(13)
District of Paraisópolis/São Paulo	22,013 interviews	9.7/1,000	8.7/1,000	0-16 years	(14)
Passo Fundo/Rio Grande do Sul	2,285 born between 2003 and 2007	6.52/1,000	5.33/1,000	0-4 years	(15)
Barra do Bugres/Mato Grosso (Semiurban region)	30,132 interviews	7.8/1,000	5.6/1,000	All ages	(16)
Pelotas/Rio Grande do Sul (Medicine Faculty at the Federal University of Pelotas)	101 interviews	—	673/1,000	12-75 years	(17)

## Methods

### Ethical Committee

The study is a retrospective study and based on the observational database analysis. The study was approved by the Ethics Committee of São Francisco University, Bragança Paulista, São Paulo, Brazil (approval #28258920.7.0000.5514).

### Patient's Selection

A retrospective study of patients with epilepsy was carried out from January 2010 to March 2021. Patients of all ages selected according to the inclusion criteria in the “Inclusion and exclusion criteria” section below were enrolled in the study. Data were collected from São Francisco University Hospital (HUSF), Bragança Paulista, located in Southeast Brazil, São Paulo. The data obtained were used for an epidemiological study of the region (rural and urban areas), which is composed of 11 municipalities served by the city of Bragança Paulista that contributes about 64.5% of the total population, according to the Brazilian Institute of Geography and Statistics (18) (Supplementary Table 1).

The selection of patients with epilepsy was performed from an electronic medical record system at the hospital, which contains all numbers of patients' medical records. We used the current International Classification of Diseases 10 (ICD 10), according to the World Health Organization (WHO), namely, G40 (G40.0, G40.1, G40.2, G40.3, G40.4, G40.5, G40.6, G40.7, G40.8, and G40.9) to define epilepsy. The study included medical records from patients from 1 January 2010 to 31 March 2021. Further, all medical records (1,272) were evaluated by two authors (Buainain, RP and Oliveira, CTP), considering anamnesis, personal and family history of epilepsy, clinical and neurological physical examination, electroencephalogram, and brain images, such as magnetic resonance imaging (MRI) and computed tomography (CT) to confirm the epilepsy diagnosis and to perform the seizures classification.

### Inclusion and Exclusion Criteria

The inclusion criteria for this study were based on the International League Against Epilepsy (ILAE) criteria: “at least two unprovoked (or reflex) seizures occurring >24 h apart” (1). Pure febrile seizures in childhood, single seizures, provoked seizures, neonatal seizures, and non-epileptic events were excluded. The febrile seizure was defined as “an event in infancy or childhood, usually occurring between 6 months and 5 years of age, associated with fever but without evidence of intracranial infection or defined cause” (19).

### Statistical Analyses

The numerical data are shown as mean ± standard deviation (SD); median (percentile 25-75). The categorical data are shown as absolute frequency and percentage. The statistical analysis was done using Mann–Whitney and Fisher's exact tests, and an alpha of 0.05 was adopted in all analyses. The statistical analysis was performed with the Statistical Package for the Social Sciences software (IBM SPSS Statistics for Macintosh, Version 27.0).

## Results

### Demographic Characteristics of the Patients With Epilepsy

The medical records of 1,272 patients were evaluated and only 618 (48.5%) patients were included in our study, according to the ILAE criteria. Bragança Paulista city presented 170,533 inhabitants and adding the comprehensive regions, it had a total population of 480,623 inhabitants, according to the IBGE, in 2020 (Supplementary Table 1). The 618 patients with epilepsy included in the present study correspond to a total prevalence ratio of 1.30 patients/1,000 habitants or an average prevalence of 11.69 patients/100,000 habitants/year.

The original cohort accounted for 618 individuals who met the criteria for defined epilepsy were 317 (51.3%) men and 301 (48.7%) women. No absolute predominance was observed (1.05:1.00) for sexes, and the percentages were nearly the same (51.3%).

### Clinical Characteristics of the Patients With Epilepsy

The clinical information is shown in Table 2. Briefly, the average age of the patients was 34.03 ± 20.66 years, and the average age at the first seizure was 15.16 ± 17.61 years. The predominant race was White in 248 (40.1%) patients; childhood febrile seizure occurred in 44 (7.9%) patients, and the family history of epilepsy in 231 (37.4%) patients. We observed focal seizures in 401 (64.9%) patients considering the seizure type. The etiology of epilepsy was structural in 254 (41.1%) patients and unknown in 238 (38.5%) patients. Most of the patients' treatments were based on anti-seizure drugs in monotherapy [389 (62.9%)], and drug-responsive occurred in 398 (64.4%) patients.

**Table 2:** Epidemiological and clinical data of 618 patients with epilepsy from a Brazilian University Hospital in Southeast São Paulo between January 2010 and March 2021.

**Marker**	Distribution (*n*, %) or numerical data^*^
**Sex**	
Male	317 (51.3%)
Female	301 (48.7%)
**Race**	
Asian	3 (0.5%)
White	248 (40.1%)
Black	12 (1.9%)
Mixed Black and White (Multiracial background)	15 (2.4%)
Not described/missing data	340 (55.0%)
**Febrile seizure**	
Absent	528 (85.4%)
Present	44 (7.9%)
Not described/missing data	41 (6.6%)
**Family history**	
Absent	226 (36.6%)
Present	231 (37.4%)
Not described/missing data	161 (26.1%)
**Seizure type**	
Focal	401 (64.9%)
Generalized	23 (3.7%)
Focal and generalized	8 (1.3%)
Not described/missing data	186 (30.1%)
**Etiology of epilepsy**	
Structural	254 (41.1%)
Genetic	3 (0.5%)
Infectious	8 (1.3%)
Metabolic	2 (0.3%)
Immune	1 (0.2%)
Unknown	238 (38.5%)
Not described/missing data	112 (18.1%)
**Epilepsy therapy**	
Monotherapy	389 (62.9%)
Polytherapy	23 (36.1%)
Not described/missing data	6 (1.0%)
**Therapy response**	
Drug responsive	398 (64.4%)
Drug-resistant	85 (13.8%)
Not described/missing data	135 (21.8%)
Age (years)	34.03 ± 20.66; 29.00 (16.00-49.00)
Age at first seizure (years)	15.16 ± 17.61; 9.00 (2.00-22.00)

* *The numerical data are shown as mean ± standard deviation (SD); median (percentile 25–75)*.

### Association Between the Demographic and Clinical Data Among Patients With Epilepsy

**Figure 1:**
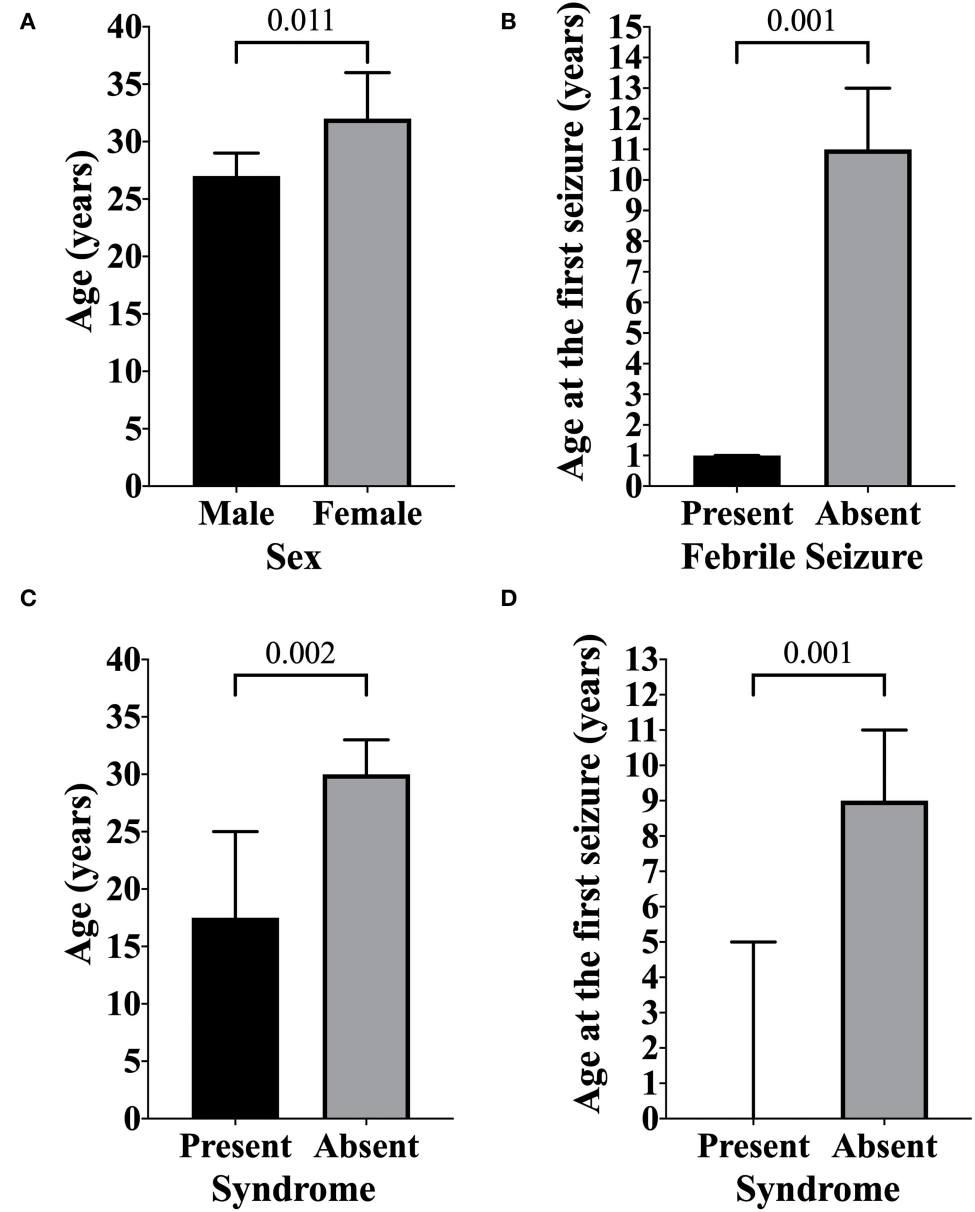
Overview of the significant association between age and sex, genetic syndrome, and first seizure. **(A)** Significant statistical data were observed when age was compared with sex (male 27 years; female 32 years; *p* = 0.011), indicating that men present seizures earlier than women. **(B)** Childhood febrile seizure was present at the age of the first seizure at ages between 0 and 2 (1 year; *p* < 0.001). In addition, the age at first seizure in patients without febrile seizures was 11 years. **(C)** Patients with any genetic syndrome presented seizures earlier (17.5 vs. 30 years; *p* = 0.002). **(D)** Age at the first seizure in patients with genetic syndromes ranged between 0 and 7 years (present <1 year; absent 9 years; *p* < 0.001). The data are shown as median and 95% confidence interval (CI). The statistical analyses were done using the Mann–Whitney test. An alpha of 0.05 was adopted in all analyses.

Significantly statistical data were observed when age (years) was compared with sex (male 32.25 ± 20.86; female 35.91 ± 20.31; *p* = 0.011) (Table 3; Figure 1A). The febrile seizure was present at the time of the first seizure at ages between 0 and 2 years (2.59 ± 5.67 years; *p* < 0.001) (Table 3; Figure 1B). Curiously, the patients without febrile seizures had an older age at the first seizure (16.63 ± 18.05 years). In addition, patients with any genetic syndrome were younger (22.31 ± 17.63 vs. 34.55 ± 20.64 years; *p* = 0.002) (Table 3; Figure 1C) and had first seizure early (5.95 ± 12.81 vs. 15.51 ± 17.68 years; *p* < 0.001) (Table 3; Figure 1D).

**Table 3:** Clinical data in association with age and age at the first seizure in patients with epilepsy from a Brazilian University Hospital in Southeast São Paulo between January 2010 and March 2021.

**Sex**	Age (years)^*^	Age at first seizure (years)^*^
Male	32.25 ± 20.86; 27 (15.00-47.50)	14.61 ± 17.54; 8 (1.00-22.25)
Female	35.91 ± 20.31; 32 (20.00-50.00)	15.74 ± 17.71; 10 (2.00-21.75)
*P*-value	0.011	0.139
**Febrile seizure**		
Absent	35.39 ± 20.73; 31 (18.00-51.00)	16.63 ± 18.05; 11 (3.00-25.75)
Present	17.71 ± 11.06; 15 (8.50-24.00)	2.59 ± 5.67; 1 (0.00-2.00)
*P*-value	<0.001	<0.001
**Syndrome**		
Absent	34.55 ± 20.64; 30 (17.00-49.00)	15.51 ± 17.68; 9 (2.00-23.00)
Present	22.31 ± 17.63; 17.5 (11.50-30.00)	5.95 ± 12.81; 0 (0.00-7.00)
*P*-value	0.002	<0.001

* *The numerical data are shown as mean ± standard deviation; median (percentile 25–75). The statistical analyses were done using the Mann–Whitney test. An alpha of 0.05 was adopted in all analyses*.

The monotherapy was commonly used among patients with generalized seizures (87.0%) followed by patients with focal seizures (56.8%) and focal and generalized seizures (50.0%) (*p* = 0.009) (Table 4).

**Table 4:** Clinical data and the type of seizure in individuals with epilepsy from a Brazilian University hospital from January 2010 to March 2021.

**Marker**	**Data**	**Focal**	**Generalized**	**Focal and generalized**	***P*-value**
Sex	Male	204 (94.9%)	8 (3.7%)	3 (1.4%)	0.250
	Female	197 (90.8%)	15 (6.9%)	5 (2.3%)	
Febrile seizure	Absent	354 (93.4%)	19 (5.0%)	6 (1.6%)	0.563
	Present	22 (91.7%)	2 (8.3%)	0 (0.0%)	
Therapy	Monotherapy	226 (56.8%)	20 (87.0%)	4 (50.0%)	0.009
	Polytherapy	172 (43.2%)	3 (13.0%)	4 (50.0%)	
Therapy response	Drug responsive	251 (77.5%)	19 (95.0%)	5 (71.4%)	0.123
	Drug resistant	73 (22.5%)	1 (5.0%)	2 (28.6%)	

*The statistical analyses were done using the Fisher's exact test. An alpha of 0.05 was adopted in all analyses*.

Table 5 presents the epilepsy etiology of all evaluated patients, being structural [male 131 (51.0%); female 12 (49.4%)] the leading cause of epilepsy. Monotherapy was statistically significant in the structural, genetic, metabolic, and unknown etiologies (*p* < 0.001); polytherapy prevailed in the infectious and immune etiologies (*p* < 0.001) (Table 5). In addition, the association between the epilepsy etiology and the resistance to treatment was significant (*p* = 0.002) (Table 5).

**Table 5:** The clinical data and etiology of epilepsy in individuals with epilepsy from a Brazilian University hospital from January 2010 to March 2021.

**Marker**	**Data**	**Structural**	**Genetic syndrome**	**Infectious**	**Metabolic**	**Immune**	**Unknown**	***P*-value**
Sex	Male	131 (51%)	2 (0.8%)	6 (2.3%)	1 (0.4%)	0 (0.0%)	117 (45.5%)	0.653
	Female	12 (49.4%)	1 (0.4%)	2 (0.8%)	1 (0.4%)	1 (0.4%)	121(48.6%)	
Therapy	Monotherapy	127 (50.4%)	2 (66.7%)	3 (37.5%)	2 (100%)	0 (0.0%)	176 (75.2%)	<0.001
	Polytherapy	125 (49.6%)	1 (33.3%)	5 (62.5%)	0 (0.0%)	1 (100%)	58 (24.8%)	
Therapy response	Drug responsive	152 (76.8%)	1 (100%)	4 (66.7%)	1 (100%)	0 (0.0%)	180 (89.1%)	0.002
	Drug resistant	46 (23.2%)	0 (0.0%)	2 (33.3%)	0 (0.0%)	1 (100%)	22 (10.9%)	

*The statistical analyses were done using the Fisher exact test. An alpha of 0.05 was adopted in all analyses*.

## Discussion

The present study aimed to describe the epidemiological profile of patients with epilepsy in a referral center in Southeast Brazil. In addition, we pointed out that since 1986, 11 epidemiological population survey studies have been conducted in Brazil (7–17) with wide variability in the epilepsy prevalence rate.

Among the studies that have been conducted in the Southeast Region of Brazil, the prevalence rates were between 9.2/1,000 and 18.6/1,000 individuals (7, 10, 11, 13, 14); in contrast, we had a prevalence rate of 1.30 cases/1,000 individuals. The contrasting results reinforce the need for an adequate epidemiological assessment of epilepsy incidence in Brazil, mainly in the Southeast region, which is the most populated region. In addition, the low prevalence of patients with epilepsy in our study can be explained by the fact that the data are from a single-center, and it cannot represent all patients with epilepsy in this part of the Southwest region of Brazil.

The epidemiological studies on epilepsy in Latin America in the last 10 years are rare. Recently, Alva-Díaz et al. (20) have published a meta-analysis in Latin America and the Caribbean, and the authors have found that the active epilepsy prevalence was 9.06 per 1,000 individuals. Melcon et al. (21) have observed a prevalence rate of 3.8 per 1,000 individuals with active epilepsy in a town in the Province of Buenos Aires.

Fiest et al. (3) demonstrated, in a meta-analysis including 222 studies between 1985 and 2017, the prevalence of active epilepsy of 6.38 per 1,000 persons. In addition, the incidence rate of epilepsy was higher in low- to middle-income countries.

Our study found a quite predominance of epilepsy in men and a mean age of patients with epilepsy of 34 years, while two previous Brazilian studies, also from the Southeast region (11, 13), have observed the predominance rate of women patients (51.2 and 51.3%), with a mean age of 36.0 and 38.4 years, respectively. In addition, a third study in the Southern region of Brazil has observed a predominance rate of mens (55.0%) with a mean age of 16.6 years (22). In the Argentine study, the prevalence rate of epilepsy was 5.3/1,000 for male patients and 7.1/1,000 for female patients (21). González et al. (23) have analyzed patients with epilepsy in Paraguay found 51.9% of male patients. Moreover, a review study considering North, Central, and South America, Europe, and Asia, between 1985 and 2007 (24) has found a higher prevalence rate of epilepsy in men than in women.

The mean age at the first seizure of the patients with epilepsy included in our study was approximately 15 years, close to a unique Brazilian study (16.6 years) in the Southern region (22). In the Argentine study, the median age at the onset of active epilepsy was 10.9 years for male patients and 16.9 years for female patients (21). According to a meta-analysis study (3), the incidence of epilepsy is higher in the youngest and oldest age groups.

In the same way, our study and Caprara et al. (22) presented a White race predominancy (40.1 and 64.2%, respectively). No previous Brazilian studies have evaluated ethnicity in patients with epilepsy. However, Szaflarski et al. (25) evaluated patients with epilepsy in Minnesota (USA), and 70.0% were categorized as White. These authors hypothesized that other ethnic minorities have more limited access to healthcare.

Further, our study observed that febrile seizure was present in 7.9% of evaluated patients with epilepsy and their age at the first seizure was ~2.6 years. Only one study in Brazil has observed the same rate; however, the authors have evaluated only patients ≥ 15 years old (22). Another Brazilian study has observed an average age of the first childhood febrile seizure of 1.6 (26) in children aged 0–5 years. Shrestha et al. (27) have analyzed the clinical characteristics of children with febrile seizures in a hospital in Nepal and found that most children (72.8%) presented their first episode of seizure below 24 months of age.

Regarding seizure classification, focal seizures were present in almost 65.0% of our patients, according to the previous Brazilian (28), American (29), and European (30) epidemiological studies. Focal seizures are the predominant type worldwide, constituting up to two-thirds of epilepsies cases (24). According to our study, González et al. (23) observed that 63.34% of patients with epilepsy presented with focal seizures. In contrast, the Argentine study has shown that seizures were generalized in 37 (58.0%) and focal in 24 (38.0%) patients with epilepsy (21). In a previous meta-analysis with 222 studies, between 1985 and 2017, the epilepsies of unknown etiology and those with generalized seizures had the highest prevalence rate (3).

Epilepsy has a variety of etiologies, ranging from genetic, metabolic, infectious, structural, immune, and unknown (31). Our study is the second that evaluated the variety of etiologies in Brazilian patients with epilepsy. The predominant etiology of epilepsy in our study was structural and unknown. A previous Brazilian study (22) has found structural and unknown etiology in 29.8 and 44.4% of the patients, respectively. Interestingly, the unknown etiology type worldwide is most prevalent, up to 50.0% (4, 24).

It was recently reported in an American study that 25–30% of new-onset seizures are provoked or secondary (32). Our study presented approximately 67.0% of the cases with infection seizures. The discrepancy might be because our referral hospital is in a low-income region, where infectious conditions, such as neurocysticercosis, human immunodeficiency virus, meningitis, and encephalitis are predominant diseases. In addition, our hospital is a tertiary center and referral center for traumatic brain injury.

Monotherapy was the most common treatment type in our evaluated patients. It was previously reported that about 50.0% of patients with epilepsy respond well to the initial monotherapy (33), while in another half, more than one antiepileptic drug is necessary (34). In our data, drug responsiveness occurred in most of the patients. Kwan et al. (35) have defined drug-resistant epilepsy “as the failure of adequate trials of two tolerated and appropriately chosen and used antiepileptic drugs (whether as monotherapies or in combination) to achieve sustained seizure freedom” and drug-responsive epilepsy as “in which the patient receiving the current antiepileptic drug regimen has been seizure-free for a minimum of three times the longest preintervention interseizure interval or 12 months, whichever is longer.” Kawn and Brodie (33) have studied 470 patients with epilepsy, of which ~50% of them responded promptly to the first monotherapy. Of the unresponsive remainder, the therapeutic strategy was to substitute or add another antiepileptic drug. Of these, only 26.0% were seizure-free, meaning that approximately 30.0% of patients will be drug-resistant. The treatment is generally pharmacological, and about 20–30% of patients are refractory to medications and thus become potential surgical candidates (36). Therefore, we achieved a successful treatment plan for those patients who are seizure-free, epilepsy controlled, and responded well to the medication.

## Conclusions

Our epilepsy prevalence rate was lower than other studies in the Southeast Region of Brazil, which reinforces the need for an adequate epidemiological assessment of epilepsy incidence in Southeast Brazil. Regarding the variables studied: sex, ethnicity, age of patients, age at onset of seizures, presence of childhood febrile seizures, type of epileptic seizure, type of treatment (mono or polytherapy), as well as drug resistance or not, our data are similar to the national and international literature. However, concerning the etiology of the seizures, our data diverged to the predominance of structural causes, whereas in studies from Brazil and the world, the unknown cause is predominant. These differences can be attributed to the characteristics of region studied by the high prevalence of neurocysticercosis and a referral center for traumatic brain injury.

## Data Availability Statement

The original contributions presented in the study are included in the article/Supplementary Material, further inquiries can be directed to the corresponding author/s.

## Ethics Statement

The studies involving human participants were reviewed and approved by Ethics Committee of São Francisco University, Bragança Paulista, São Paulo, Brazil (approval #28258920.7.0000.5514). Written informed consent for participation was not provided by the participants' legal guardians/next of kin because: only medical record review.

## Author Contributions

RB, FM, and MO wrote the manuscript. RB and CO performed the clinical research. FM performed all statistical analyses and designed the figures. MO designed the research. All authors have read and approved the final manuscript.

## Conflict of Interest

The authors declare that the research was conducted in the absence of any commercial or financial relationships that could be construed as a potential conflict of interest.

## Publisher's Note

All claims expressed in this article are solely those of the authors and do not necessarily represent those of their affiliated organizations, or those of the publisher, the editors and the reviewers. Any product that may be evaluated in this article, or claim that may be made by its manufacturer, is not guaranteed or endorsed by the publisher.

## Abbreviations

IBGE: brazilian institute of geography and statistics
CT: computed tomography
CI: confidence interval
ICD 10: international classification of diseases 10
ILAE: international league against epilepsy
LTE: lifetime epilepsy
MRI: magnetic resonance imaging
USF: são francisco university
HUSF: são francisco university medical school hospital
GBDE: the global burden of disease study of epilepsy
USA: united states of america
WHO: world health organization.
