# Supplementary material for: Epidemiologic Profile of Patients With Epilepsy in a Region of Southeast Brazil: Data From a Referral Center
Source: Front Neurol. 2022 May 10;13:822537. doi: 10.3389/fneur.2022.822537 (PMC9128524; doi:10.3389/fneur.2022.822537)
Supplement: Supplementary file 1 [file Table_1.docx]

**Supplementary Table 1.** Cities that belong to the regional health of Bragança Paulista, São Paulo state, Brazil, and the total population and patients with epilepsy are included in the study. Data source: IBGE, July 23, 2021.

| **Cities** | **Population number** | **Patients number** |
| --- | --- | --- |
| Atibaia | 144,088 | 94 |
| Bom Jesus dos Perdões | 25,985 | 42 |
| Bragança Paulista | 170,533 | 214 |
| Joanópolis | 13,338 | 16 |
| Nazaré Paulista | 18,698 | 23 |
| Pedra Bela | 6,110 | 32 |
| Pinhalzinho | 15,388 | 40 |
| Piracaia | 27,462 | 45 |
| Socorro | 41,352 | 31 |
| Tuiuti | 6,977 | 18 |
| Vargem | 10,692 | 23 |
| Outside the coverage area | --- | 19 |
| Missing data | --- | 21 |
| Total | 480,623 | 618 |
